# Colitis aggravated by *Mrgprb2* knockout is associated with altered immune response, intestinal barrier function and gut microbiota

**DOI:** 10.1113/EP090635

**Published:** 2022-11-28

**Authors:** Ming Shao, Jingwen Liu, Hesheng Luo

**Affiliations:** ^1^ Department of Gastroenterology Renmin Hospital of Wuhan University Wuhan China; ^2^ Department of Gastroenterology Hubei Key Laboratory of Digestive Diseases Wuhan China

**Keywords:** gut microbiota, inflammatory cells, intestinal barrier, mast cells, mrgprb2, ulcerative colitis

## Abstract

Ulcerative colitis (UC) is a chronic immune‐related disease, and changes in the intestinal microbiota and damage to the intestinal barrier contribute to its pathogenesis. Mast cells (MCs) are widely distributed in the gastrointestinal tract and are thought to be related to the pathogenesis of UC. Human mas‐related G protein‐coupled receptor X2 (MRGPRX2) and its mouse homologue, Mrgprb2, are selectively expressed on MCs to recruit immune cells and modulate host defence against microbial infection. To investigate the role of Mrgprb2 in UC in mice, we compared the differences between *Mrgprb2* knockout (b2KO) male mice and wild‐type (WT) male mice with dextran sulfate sodium (DSS)‐induced colitis in the severity of clinical symptoms, inflammatory cell infiltration, degree of intestinal barrier damage and composition of the intestinal flora. The results showed that weight loss, disease activity index score, colon shortening and colonic pathological damage were significantly increased in b2KO mice while MC activation, cytokine and chemokine secretion, and inflammatory cell infiltration were decreased. In addition, the abundance and diversity of the intestinal microbiota were reduced in b2KO mice. B2KO mice also exhibited a reduction of probiotics such as norank_f_Muribaculaceae and *Lactobacillus* and increase of harmful bacteria like *Escherichia*–*Shigella*. Intestinal mucosal barrier damage of b2KO mice was more severe than that of WT mice due to the attenuated expression of mucin‐2 and occludin. These results demonstrated that MRGPRB2 may have a protective effect on DSS‐induced colitis by altering the intestinal flora, participating in barrier repair and recruiting inflammatory cells to eliminate pathogens.

## Abstract (highlights)

- **What is the central question of this study?**
  What is the role of mas‐related G protein‐coupled receptor X2 (MRGPRX2/Mrgprb2) in ulcerative colitis in relation to the intestinal flora, intestinal barrier and immune response?
- **What is the main finding and its importance?**
  Knockout of mouse *Mrgprb2* aggravates dextran sulfate sodium (DSS)‐induced colitis, which is associated with altered gut microbiota and immune response and disruption of the intestinal barrier. MRGPRB2 may have a protective effect on DSS‐induced colitis.

## 1 INTRODUCTION

Ulcerative colitis (UC) is a kind of chronic inflammatory bowel disease (IBD) initiating in the rectum and colon (Kobayashi et al., 2020). Multiple factors including genes, environment, immune system and gut microbiota closely interact with each other, and changes to one factor can affect intestinal homeostasis based on a delicate equilibrium and lead to the occurrence of diseases such as UC (De Zuani et al., 2018; Feuerstein et al., 2019; Kudelka et al., 2020). Increasing emerging evidence supports the assumption that mast cells (MCs), of all the factors that affect intestinal homeostasis, play an important role (Albert‐Bayo et al., 2019; De Zuani et al., 2018; Lyons & Pullen, 2020).

MCs are widely distributed in the gastrointestinal tract, and numerous intestinal diseases including UC are thought to be related to the role of uncontrolled or disordered MC activation (Redegeld et al., 2018). Upon stimulation, MCs release proteases, cytokines, chemokines, growth factors and mitogens to adjust vascular and epithelial permeability, tissue repair, innate and adaptive immunity, and bacterial clearance, which is important for maintaining intestinal homeostasis (Gupta & Harvima, 2018; Yu et al., 2016). Degranulation and cytokine release of MCs are induced by activation of various cell surface receptors such as high‐affinity immunoglobulin E (IgE) receptor (FcεRI) and G‐protein‐coupled receptors (GPCRs) (Bulfone‐Paus et al., 2017; Meixiong & Dong, 2017). Recently, researchers have begun to focus on mas‐related G protein‐coupled receptor X2 (MRGPRX2), which activates MCs through a non‐IgE mechanism and was found to be only expressed in MCs and dorsal root ganglia (Kajiwara et al., 2010; Meixiong & Dong, 2017; Tatemoto et al., 2006).

Human MRGPRX2 and the mouse homologue, Mrgprb2, are specifically expressed on MCs (Kumar et al., 2021). Studies showed that MRGPRX2/Mrgprb2 mediates neurogenic inflammation. Substance P (SP)‐mediated MC activation via MRGPRX2 led to recruitment of innate immune cells to the injury site and the release of multiple pro‐inflammatory cytokines and chemokines which induce inflammatory mechanical and thermal hyperalgesia. Mrgprb2‐deficient mice had a striking decrease in recruitment of immune cells at the injured site (Green et al., 2019). MRGPRX2/Mrgprb2 also has an effect on the immune response to bacterial infections. Competence‐stimulating peptide (CSP)‐1, one of the Gram‐positive quorum‐sensing molecules, activated MCs through Mrgprb2 and MRGPRX2 and led to inhibition of bacterial growth and biofilm formation (Pundir et al., 2019). In models of nasopharynx, peritoneum and skin infection, Mrgprb2‐deficient mice showed reduced bacterial clearance, while activation of Mrgprb2 eliminated bacteria and improved disease scores (Pundir et al., 2019). At present, researchers have found some associations between MRGPRX2 and UC; for example, in UC patients, expression of the proteolytic precursors of the MRGPRX2 agonist was increased in inflamed UC compared to uninflamed UC (Chen et al., 2021). A new therapeutic target may be obtained by studying the effect of Mrgprb2 on UC, and related studies are still insufficient.

The aim of this work is to investigate the effect of MRGPRX2/Mrgprb2 on UC and explore the mechanism through which MRGPRX2/Mrgprb2 participates in the pathogenesis of UC.

## 2 METHODS

### 2.1 Ethical approval

Animal experiments were approved by the Animal Care and Ethics Committee of Wuhan University (Approval No. 20210303) in accordance with the *Guide for the Care and Use of Laboratory Animals* published by the National Institutes of Health (2011, 8th edition). The investigators understand and complied with principles and standards for reporting animal experiments in *Experimental Physiology* and have taken all steps to minimize animals’ pain and suffering (Grundy, 2015).

### 2.2 Animals and dextran sulfate sodium induced colitis

Six‐ to eight‐week‐old male B2KO C57BL/6J mice were purchased from Gem Pharmatech Co., Ltd (Jangsu, China). Six‐ to eight‐week‐old female C57BL/6J WT mice were purchased from Beijing Vital River Laboratory Animal Technology Co., Ltd (Beijing, China). Mice were mated, bred and raised in specific pathogen free (SPF) environment in Renmin Hospital of Wuhan University Animal Experiment Center. Homozygous b2 KO male mice and WT mice were screened by genetic identification from the progeny. Thirty‐two male C57BL/6J mice (6–8 weeks old) were randomly assigned to two control groups (*n* = 8 mice/group) that received no dextran sulfate sodium (DSS), namely the control group and b2KO group, and two DSS‐treated groups (*n* = 8 mice/group), namely the DSS group and b2KODSS group. Mice were raised under a 12 h–12 h dark–light cycle with an ambient temperature of 22 ± 1°C, 50 ± 10% humidity, and with free access to food and water. After 1 week of adaptive feeding, mice of the two DSS‐treated groups were given 3% DSS (36–50 kDa; MP Biomedicals, Santa Ana, CA, USA) in the drinking water for 7 days to induce acute colitis and the control groups received normal water. Body weight, the stool consistency and haematochezia were recorded daily to determine the disease activity index (DAI) (Murthy et al., 1993). At day 8 the mice were anaesthetized with 5% isoflurane gas until the level of surgical anaesthesia was reached. The mice were then killed without consciousness by cervical dislocation. Their intestinal tracts were removed to measure the length and collect the intestinal contents. The distal colon was fixed with 4% paraformaldehyde for haematoxylin–eosin (H&E) staining, and the rest was cryopreserved in liquid nitrogen for other tests.

### 2.3 Histological evaluation of colon sections

The colon tissues fixed with paraformaldehyde were embedded in paraffin wax and a 4 μm section of each sample was placed on a glass slide and stained with H&E. Histological damage scores for H&E stained colon sections were assessed according to published guidelines: (a) crypt distortion and loss (normal to severe, 0−3), (b) inflammatory cell infiltration (normal to intensive, 0−3), (c) muscle thickening (presence of significant muscle thickening, 0−3), (d) goblet cell depletion (absence and presence, 0−1), and (e) crypt abscess (absence and presence, 0−1) (Cooper et al., 1993). Five visual fields were selected for each section and evaluated by an experienced pathologist in a blinded manner.

### 2.4 Immunofluorescence of intestinal barrier related proteins

Paraffin‐embedded colon sections were deparaffinized and rehydrated through a series of xylene and ethanol washes. After washing with phosphate‐buffered saline (PBS), sections were blocked in 10% goat serum in PBS for 1 h in the dark in a humid chamber at room temperature and then incubated overnight with rabbit anti‐occludin antibody (1:100) (Abcam, Waltham, MA, USA) and anti‐mucin2 (MUC2) antibody (1:1000) (Abcam). Tissue sections were then incubated with secondary antibody for Alexa‐Fluor 488 donkey anti‐Rabbit IgG (Thermo Fisher Scientific, Waltham, MA, USA) in the dark at room temperature for 50 min. Then 4′,6‐diamidino‐2‐phenylindole (DAPI) was add to the slides for incubation in the dark at room temperature for 10 min. Finally, the slides were washed three times in PBS and mounted with Antifade Mounting Medium and stored at 4°C in the dark until imaging.

### 2.5 Measurement of relative mRNA expression in colon tissues

Total RNA was isolated using TRIzol reagent (Servicebio, Wuhan, China). The supernatant was removed after centrifugation. The RNA was reverse‐transcribed into cDNA by using the Servicebio RT First Strand cDNA Synthesis Kit in a 20 μl reaction volume. Then cDNA samples were subjected to quantitative real time PCR using CFX Opus Real‐Time PCR System (Bio‐Rad Laboratories, Hercules, CA, USA) and SYBR Green qPCR Master Mix (no ROX; Servicebio) according to the manufacturer's directions. After the PCR reaction system was configured, amplification was carried out according to the steps of 95°C for 10 min, 40 cycles, 95°C for 15 s and 60°C for 30 s. During the temperature rise from 65°C to 95°C, a fluorescence signal was collected every 0.5°C. The results were analysed by the $2^{-\Delta \Delta C_{T}}$ method. The primers in this experiment are listed in Table 1.

**TABLE 1:** Details of primer sequences used for the study

Name	Sequence (5′ → 3′)	Target products
M‐GAPDH‐S	CCTCGTCCCGTAGACAAAATG	GAPDH
M‐GAPDH‐A	TGAGGTCAATGAAGGGGTCGT	
M‐mpo‐S	CAATGTCTTCACCAACGCTTTC	MPO
M‐mpo‐A	AATGCCACCTTCCAACACGA	
M‐Mcpt4‐S	CTTCTATTCCAATCTCCATGACATC	mMCP4
M‐Mcpt4‐A	AGGTTCTGTCACTCCAGTTCGC	
M‐Tpsb2‐S	TGGATACATTTCTGCGGAGGTT	mMCP6
M‐Tpsb2‐A	GACATTCACAGGGACCTCAAGC	
M‐ZO1‐S	GGGAAAACCCGAAACTGATG	ZO‐1
M‐ZO1‐A	GCTGTACTGTGAGGGCAACG	
M‐claudin3‐S	GAGTGCTTTTCCTGTTGGCG	claudin3
M‐claudin3‐A	GTAGTCCTTGCGGTCGTAGG	
M‐Cdh1‐S	ATGAAGGCGGGAATCGTG	E‐cadherin
M‐Cdh1‐A	CCGTAGAAACAGTAGGAGCAGC	

### 2.6 Analyses of inflammatory cytokines in colon tissues

The colon tissues were accurately weighed and ground into homogenate in 0.9% saline solution under in an ice bath. After centrifugation, the supernatant was taken for determination. The levels of tumour necrosis factor‐α (TNF‐α), interleukin (IL)‐1β, IL‐6, IL‐10, chemokines‐CCL2, CCL3, granulocyte colony‐stimulating factor (GM‐CSF), SP, nerve growth factor (NGF) and histamine in colon tissues were measured with enzyme‐linked immunosorbent assay (ELISA) kits (Multisciences (Lianke) Biotech, Co., Ltd, Hangzhou, China), and specific experimental steps were carried out according to the instructions. For each sample, wells were designed in triplicate and the average value was finally taken.

### 2.7 Analysis of colonic contents by 16S RNA gene sequencing

Colon contents of mice were collected and rapidly frozen with liquid nitrogen and then stored at −80°C. Microbial community genomic DNA was extracted using the E.Z.N.A. soil DNA kit (Omega Bio‐tek, Norcross, GA, USA) according to manufacturer's instructions. The DNA quality was checked with 1% agarose gel electrophoresis and the concentration and purity of the obtained DNA were determined with a Nano Drop 2000 UV‐Vis spectrophotometer (Thermo Fisher Scientific). The V3−V4 hypervariable regions of the bacterial 16S rRNA gene were amplified by an ABI Gene Amp 9700 PCR thermocycler (Thermo Fisher Scientific) with primers 338F (5′‐ACTCCT ACGGGAGGCAGCAG‐3′) and 806R (5′‐GGACTACHVGGGTWTCTAAT −3′). After PCR amplification and purification, PCR products were quantified and homogenized using a Picogreen dye fluorometer, and sequenced on an Illumina MiSeq PE300 platform/NovaSeq PE250 platform (Illumina, San Diego, CA, USA).

### 2.8 Statistical analyses

All experimental data were analysed and visualized using GraphPad Prism version 9.20 for Windows (GraphPad Software, San Diego, CA, USA) and presented as the mean ± standard deviation. The Shapiro–Wilk test was used to test for normal distribution of numerical data. Data conforming to normal distribution were assessed with one‐way ANOVA followed by a *post hoc* Bonferroni comparison for multiple groups. Analysis of histological scores between the DSS group and b2KODSS group was performed with the Mann–Whitney test. Analysis of microbiota at the phylum and genus level was by two‐way ANOVA followed by a *post hoc* Bonferroni comparison for multiple groups. A *P*‐value of <0.05 was considered statistically significant.

## 3 RESULTS

### 3.1 B2KO mice expressed more severe symptoms

**FIGURE 1:**
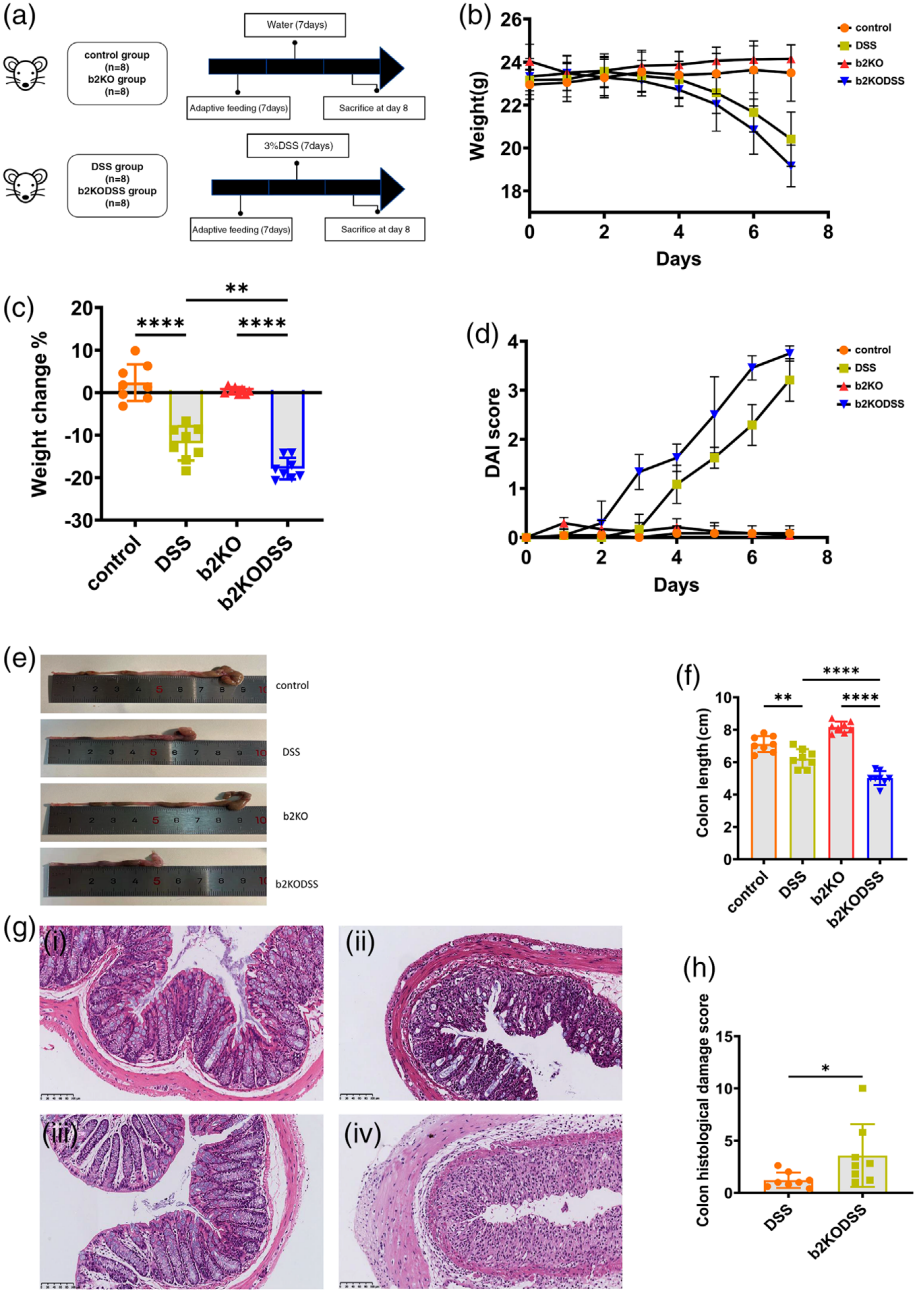
Lack of Mrgprb2 aggravated symptoms of DSS‐induced colitis in mice. (a) Diagram showing the animal experimental design. (b) Daily mouse body weight. (c) Loss of body weight. (d) DAI score. (e, f) Colon length. (g) Representative H&E images of colon sections for (i) control group, (ii) DSS group, (iii) b2KO group, and (iv) b2KODSS group. Scale bar, 100 μm. (h) Histological scores. All data are expressed as means ± SD (*n* = 8 mice/group). Statistical significance was indicated as follows: ^*^ *P* < 0.05, ^**^ *P* < 0.01 and ^****^ *P* < 0.0001.

DSS added to drinking water induced colitis in mice, resembling the pathogenesis of human UC. We evaluated clinical symptoms of colitis in b2KO mice and wild‐type mice. After 7 days of 3% DSS treatment, both groups of mice suffered severe colonic inflammation and the body weight of mice in both DSS treatment groups was decreased at the end of the experiment (Figure 1a, b). We observed that mice in the b2KODSS group began to lose weight from day 3 and the loss in the next few days was greater than in the DSS group (*P* = 0.006) (Figure 1c). Colon length and DAI score are important indicators of the severity of colon inflammation. Compared with the DSS group, the b2KODSS group mice had significantly shorter colons (*P* < 0.0001) and higher DAI score (Figure 1d–f). We then performed a pathological analysis of the colon tissue and found that in the two control groups, the colonic mucosa, submucosa, muscular layer and outer membrane were intact. However, the colonic tissue sections of the two experimental groups treated with DSS exhibited crypt distortion, goblet cell loss, inflammatory cell infiltration and severe mucosal damage, and the b2KODSS group had more severe colonic injury (*P* = 0.0138) (Figure 1g, h). These results suggested that the clinical manifestations of DSS‐induced colitis in mice were more severe when Mrgprb2 was deficient.

### 3.2 B2KO mice had lower myeloperoxidase expression and exhibited attenuated MC degranulation and cytokine expression

**FIGURE 2:**
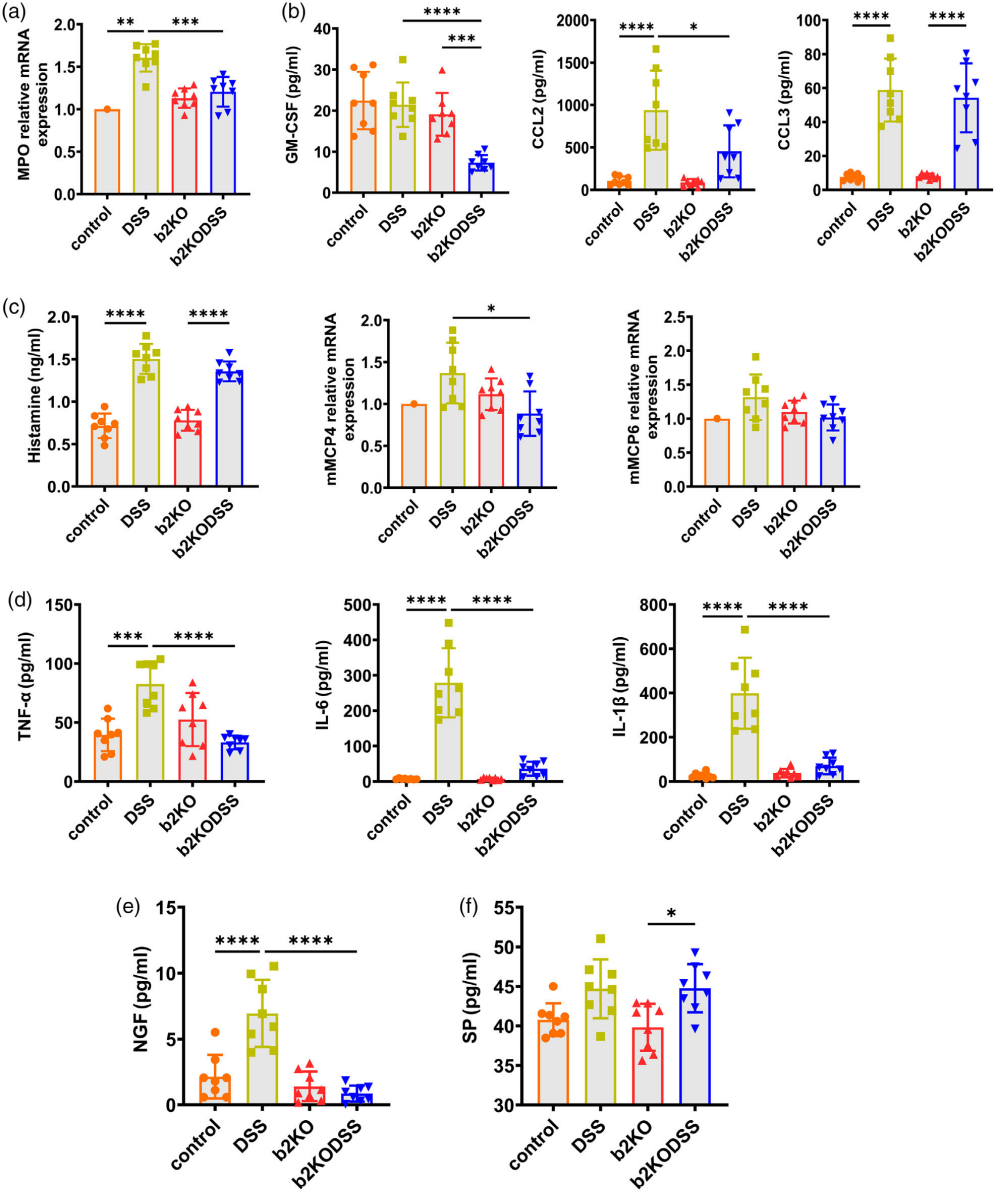
Mrgprb2 deficiency attenuates MPO expression, MC activation and cytokine secretion. (a) The colonic mRNA expression levels of MPO. (b) ELISA analysis of GM‐CSF, CCL2 and CCL3. (c) ELISA analysis of histamine, and the colonic mRNA expression levels of mMCP4 and mMCP6. (d) ELISA analysis of TNF‐α, IL‐6 and IL‐1β. (e) ELISA analysis of NGF. (f) ELISA analysis of SP. All data are expressed as means ± SD (*n* = 8 mice/group). Statistical significance was indicated as follows: ^*^ *P* < 0.05, ^**^ *P* < 0.01, ^***^ *P* < 0.001 and ^****^ *P* < 0.0001.

As Mrgprb2 is a major driver of the recruitment of inflammatory cells (Subramanian et al., 2016), we hypothesized that b2KO mice would have a reduced ability to recruit inflammatory cells to the site of injury. We tested the colonic mRNA expression levels of myeloperoxidase (MPO) and found that MPO expression increased in both DSS treatment groups while the expression of MPO in the b2KODSS group was lower than that in the DSS group (*P* = 0.0002) (Figure 2a). In addition, the levels of GM‐CSF (*P* < 0.0001) and CCL2 (*P* = 0.0108) in the b2KODSS group were also lower than in the DSS group (Figure 2b), which might suggest that b2KO mice recruited fewer inflammatory cells. Histamine levels, mouse mast cell protease (mMCP) 4 and mMCP6 in all four groups were analysed to measure the mast cell activation. Histamine level and the mRNA expression level of mMCP6 in the b2KODSS group were lower than in the DSS group, although the differences were not statistically significant. The mRNA expression level of mMCP4 in the b2KODSS group was lower than that in DSS group (*P* = 0.0146) (Figure 2c). We tested the levels of inflammatory factors in intestinal samples including TNF‐α, IL‐6 and IL‐1β. As shown in Figure 2d, the expression of TNF‐α, IL‐6 and IL‐1β in the b2KODSS group was significantly lower than that in DSS group (*P* < 0.0001). The Mrgprb2 receptor plays an important role in the process of the interaction between MCs and nerves (Green et al., 2019). We verified NGF and SP expression levels. The expression of NGF in the b2KODSS group was reduced (*P* < 0.0001) (Figure 2e). The SP level was not significantly different between the two groups (Figure 2f).

### 3.3 Damage to the colonic mucosal barrier was more serious in b2KO mice

**FIGURE 3:**
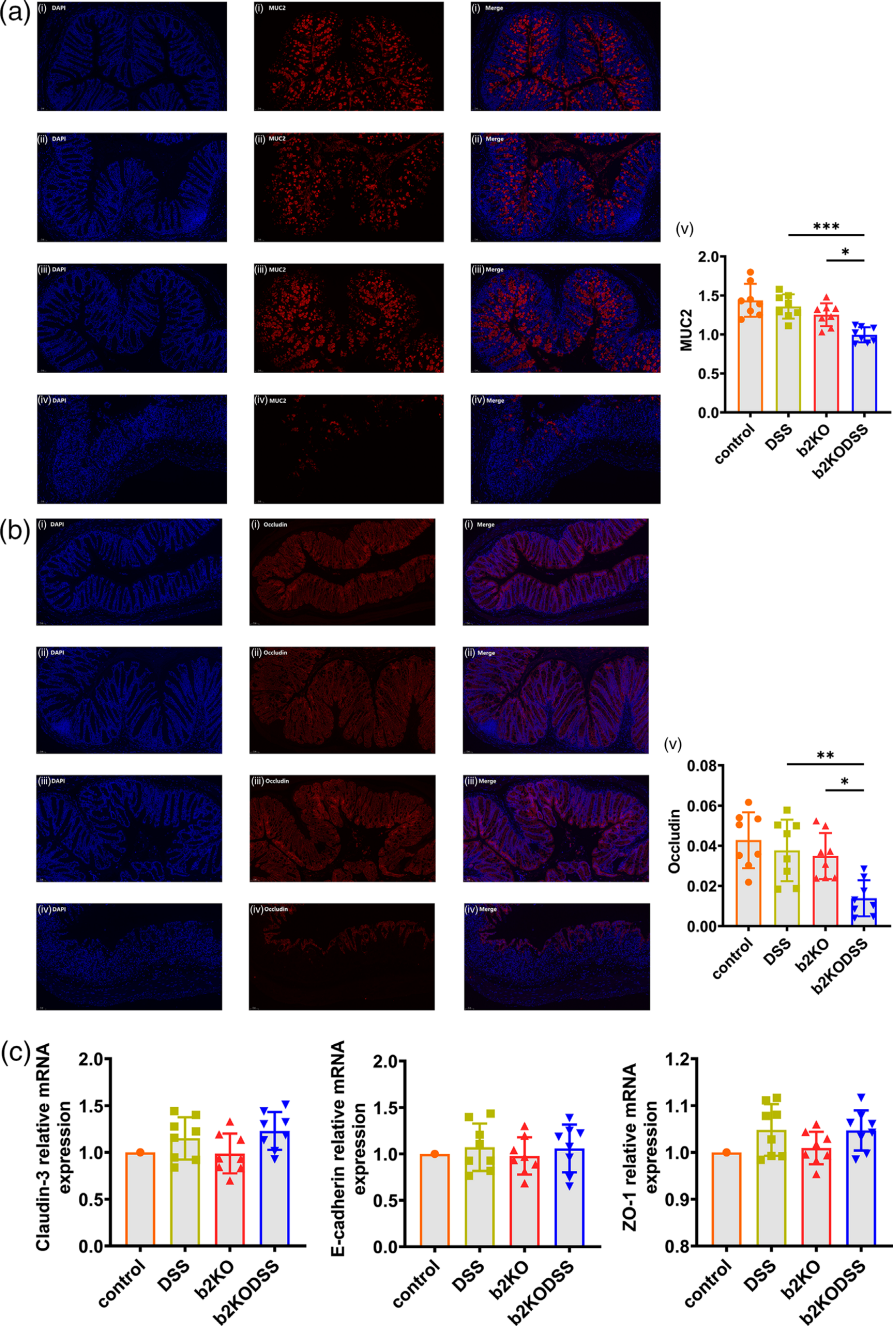
b2KO mice expressed fewer mucins and TJ proteins. (a) Immunofluorescence analysis on MUC2 in colon sections: (i) control group, (ii) DSS group, (iii) b2KO group, (iv) b2KODSS group, (v) average fluorescence intensity of MUC2. Representative images are shown. Scale bar, 50 μm. (b) Immunofluorescence analysis on occludin in colon sections: (i) control group, (ii) DSS group, (iii) b2KO group, (iv) b2KODSS group, (v) average fluorescence intensity of occludin. Representative images are shown. Scale bar, 50 μm. (c) The colonic mRNA expression levels of ZO‐1, E‐cadherin, claudin‐3. All data are expressed as means ± SD (*n* = 8 mice/group). Statistical significance was indicated as follows: ^*^ *P* < 0.05, ^**^ *P* < 0.01 and ^***^ *P* < 0.001.

In the colon of UC patients, various factors lead to decreased synthesis of colonic mucin, especially MUC2, and altered expression of tight junction (TJs) protein, which ultimately leads to impaired intestinal barrier function (D'Alessio et al., 2021; Kobayashi et al., 2020). Immunofluorescence examination showed that the expression of MUC2 (*P* < 0.0001) and occludin (*P* = 0.0046) in the b2KODSS group was weaker than that in the DSS group (Figure 3a, b). The mRNA expression levels of zonula occludens‐1 (ZO‐1), E‐cadherin and claudin‐3 were not significantly different between the DSS group and b2KODSS group (Figure 3c).

### 3.4 The composition of the gut microbiota was significantly changed in b2KO mice

**FIGURE 4:**
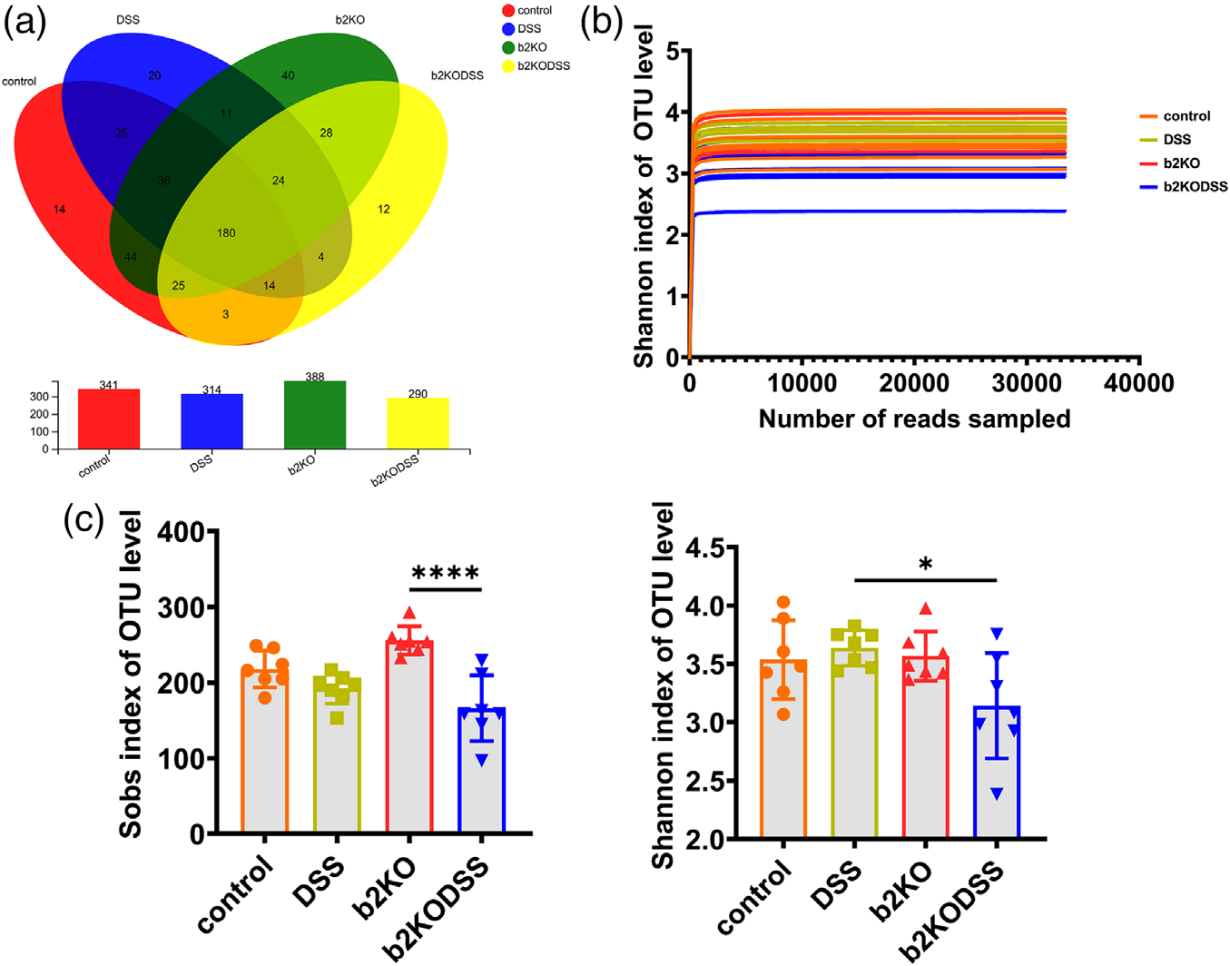
b2KO decreased the gut microbiota abundance. (a) Venn diagram based on OTU level. (b) Rarefaction curves based on the OTU level. (c) Sobs and Shannon indices of OTU level. All data are expressed as means ± SD (*n* = 7 mice/group). Statistical significance was indicated as follows: ^*^ *P* < 0.05 and ^****^ *P* < 0.0001.

Mrgprb2 contributes to host defence in mice and induces protective immunity against bacterial infection (Pundir et al., 2019). Previous studies have suggested that intestinal microbiota changes play an important role in the pathogenesis of IBD (Imhann et al., 2018; Pundir et al., 2019). In order to verify whether b2KO had an effect on the diversity of intestinal microbes in mice, we conducted a 16S RNA study on the collected intestinal contents. As expected, the number of operational taxonomic units (OTUs) in both DSS treatment groups was significantly lower than that in both control groups, and the b2KODSS group had the lowest OTU level. The characteristics and common taxa of the four groups were determined by using a Venn diagram, which revealed that 180 OTUs coexisted in the four groups and the number of unique OTUs in the control group, DSS group, b2KO group and b2KODSS group was 14, 20, 40 and 12, respectively (Figure 4a). The rarefaction curve of observed OTUs plateaued with the current sequencing, which indicated that the sequencing depth was sufficient to reflect the diversity information of the sample, and the sequencing result was credible (Figure 4b). Then the α‐diversity of the samples was analysed, and according to the Sobs index we found the community abundance of the two DSS treatment groups decreased compared with the two control groups. The Shannon index, which reflects community diversity, declined in the b2KODSS group indicating that species diversity of the b2KODSS group was low (*P* = 0.0391) (Figure 4c).

The species abundance of each sample was counted at the phylum level, and the community composition was studied using a bar graph (Figure 5a). According to the analysis, a total of eight phyla were detected, among which Bacteroidetes, Firmicutes, Proteobacteria and Actinobacteriota accounted for the largest proportion. Compared with the two control groups, the content of Proteobacteria in the samples of the two DSS treatment groups was significantly increased. Among the four groups, the b2KODSS group had the lowest proportion of Firmicutes and the highest proportion of Proteobacteria (Figure 5b). The comparison between the DSS group and b2KODSS group showed that the proportion of Proteobacteria in the samples of the b2KODSS group was higher (Figure 5c). At the genus level, the dominant bacteria in each group were norank_f_Muribaculaceae, which has been shown to improve intestinal mucositis in mice (Imhann et al., 2018) (Figure 5d). Norank_f_Muribaculaceae decreased in both DSS treatment groups, and the reduction was more significant in the b2KODSS group. Another beneficial bacterium, *Lactobacillus*, was also strikingly reduced in both DSS treatment groups (Figure 5e). The proportion of the harmful bacteria *Escherichia*–*Shigella* was elevated in the DSS group and b2KODSS group, and higher in the b2KODSS group (Figure 5f).

FIGURE 5Knockout of *Mrgprb2* changed the composition of gut microbiota. (a) Composition of microbiota in mice at the phylum level. (b) Analysis of microbiota in four group at the phylum level. (c) Differences of microbiota between DSS group and b2KODSS group at the phylum level. (d) Composition of microbiota in mice at the genus level. (e) Analysis of microbiota in four group at the genus level. (f) Differences of microbiota between DSS group and b2KODSS group at the genus level. All data are expressed as means ± SD (*n* = 7 mice/group). Statistical significance was indicated as follows: ^*^ *P* < 0.05, ^**^ *P* < 0.01, ^***^ *P* < 0.001 and ^****^ *P* < 0.0001.

**Figure:**
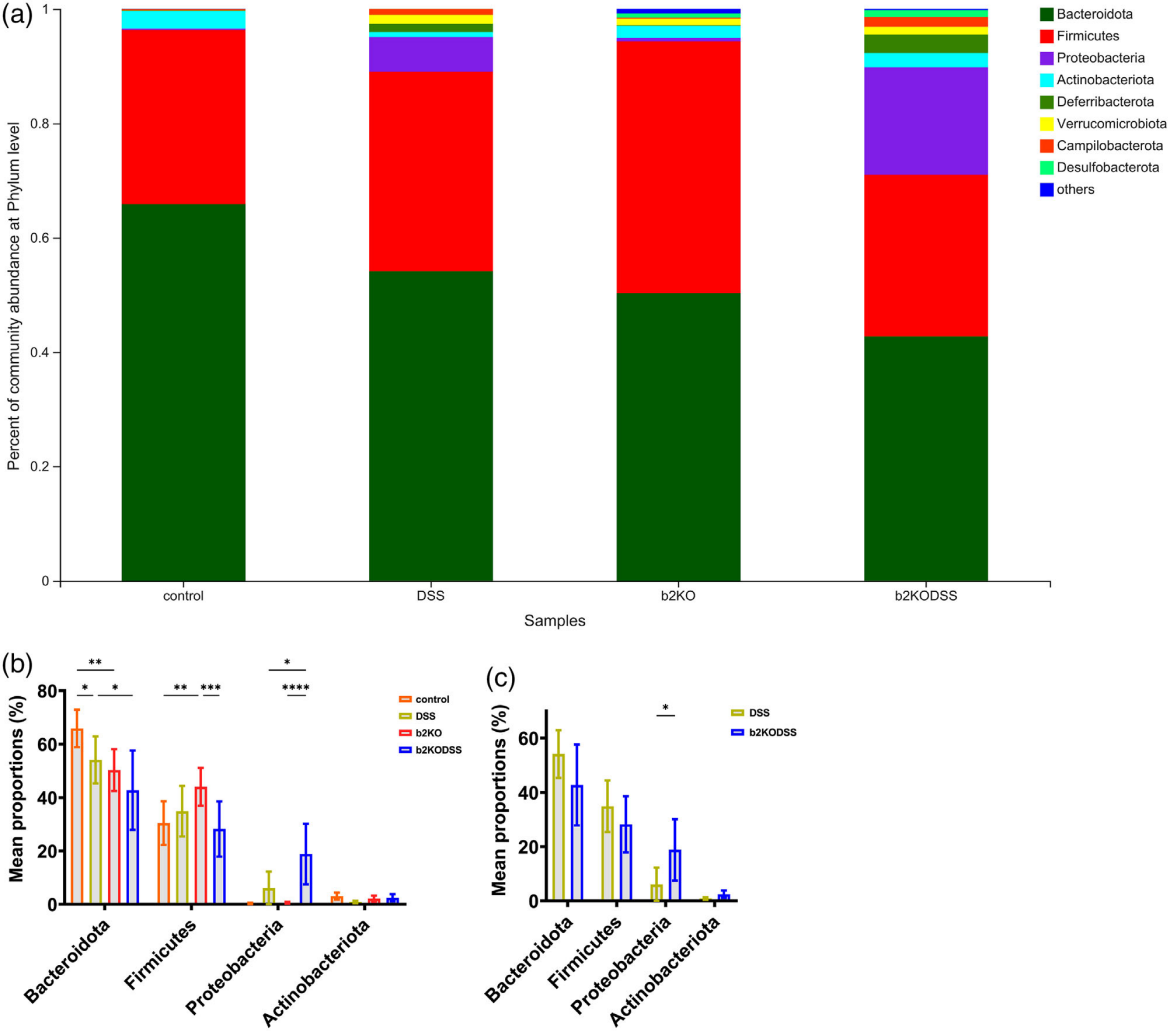

**Figure:**
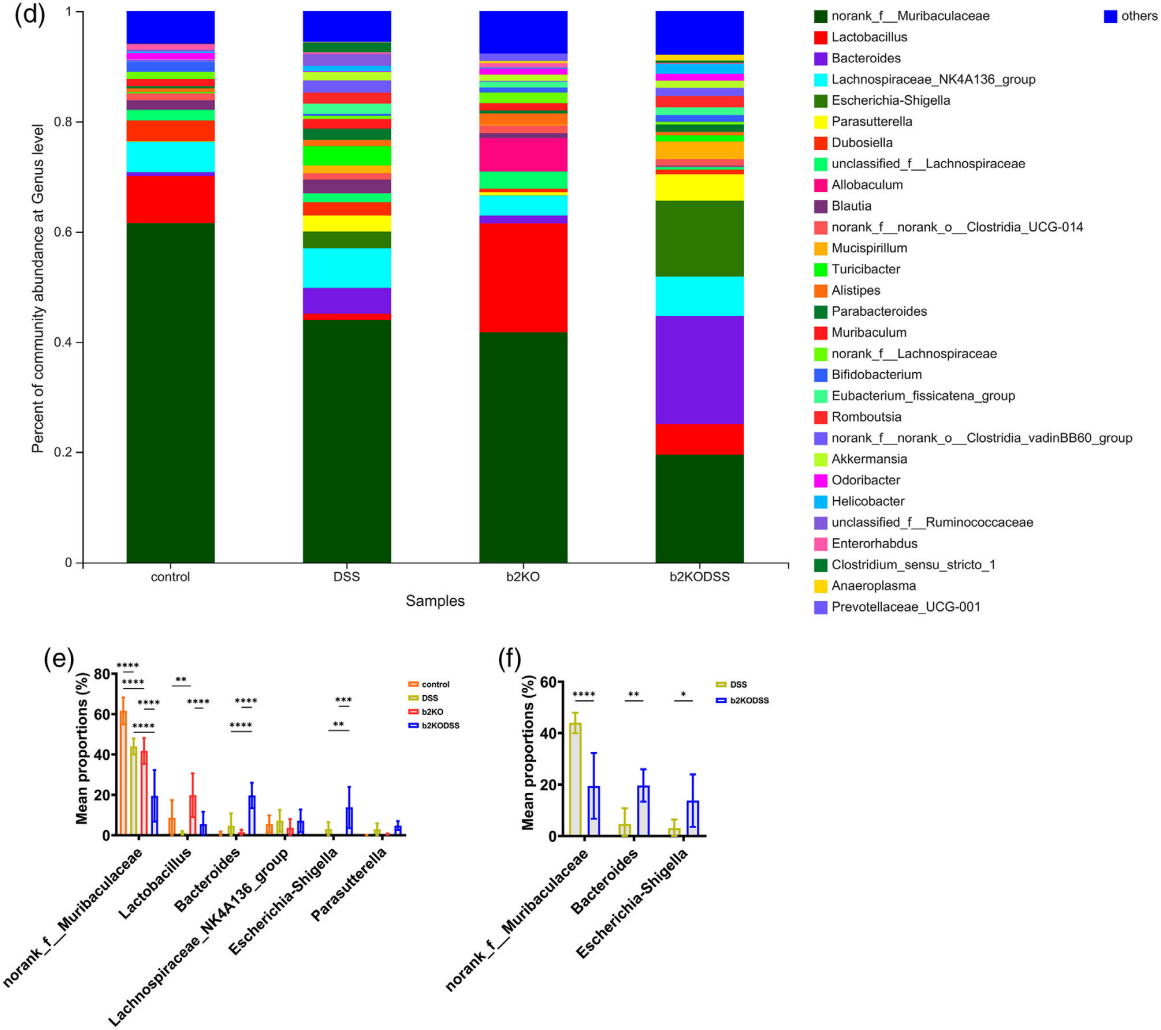

**FIGURE 6:**
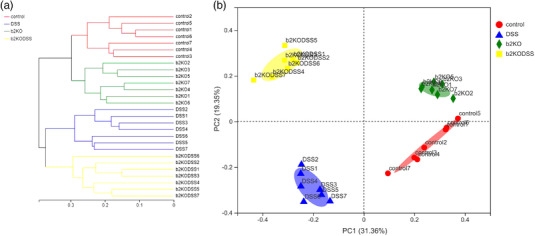
β‐Diversity analysis of gut microbiota. (a) Hierarchical cluster analysis. (b) PCoA plot on OTU level based on the Bray–Curtis index

β‐Diversity analysis is an inter‐group comparative analysis of species diversity in different habitats or microbial communities to explore the similarity or difference in community composition among different groups. A hierarchical cluster analysis at the OTU level showed that there were significant differences in microbial community structure between the two experimental groups and the two control groups (Figure 6a), and the bacterial composition in the groups was similar. Principal coordinates analysis (PCoA) is the projection of a sample distance matrix through different distance algorithms. A PCoA analysis of the four groups of samples at the OTU level showed that DSS treatment significantly altered the intestinal microbial structure, and knockout of *Mrgprb2* gene also affected the intestinal microbiota (Figure 6b).

## 4 DISCUSSION

**FIGURE 7:**
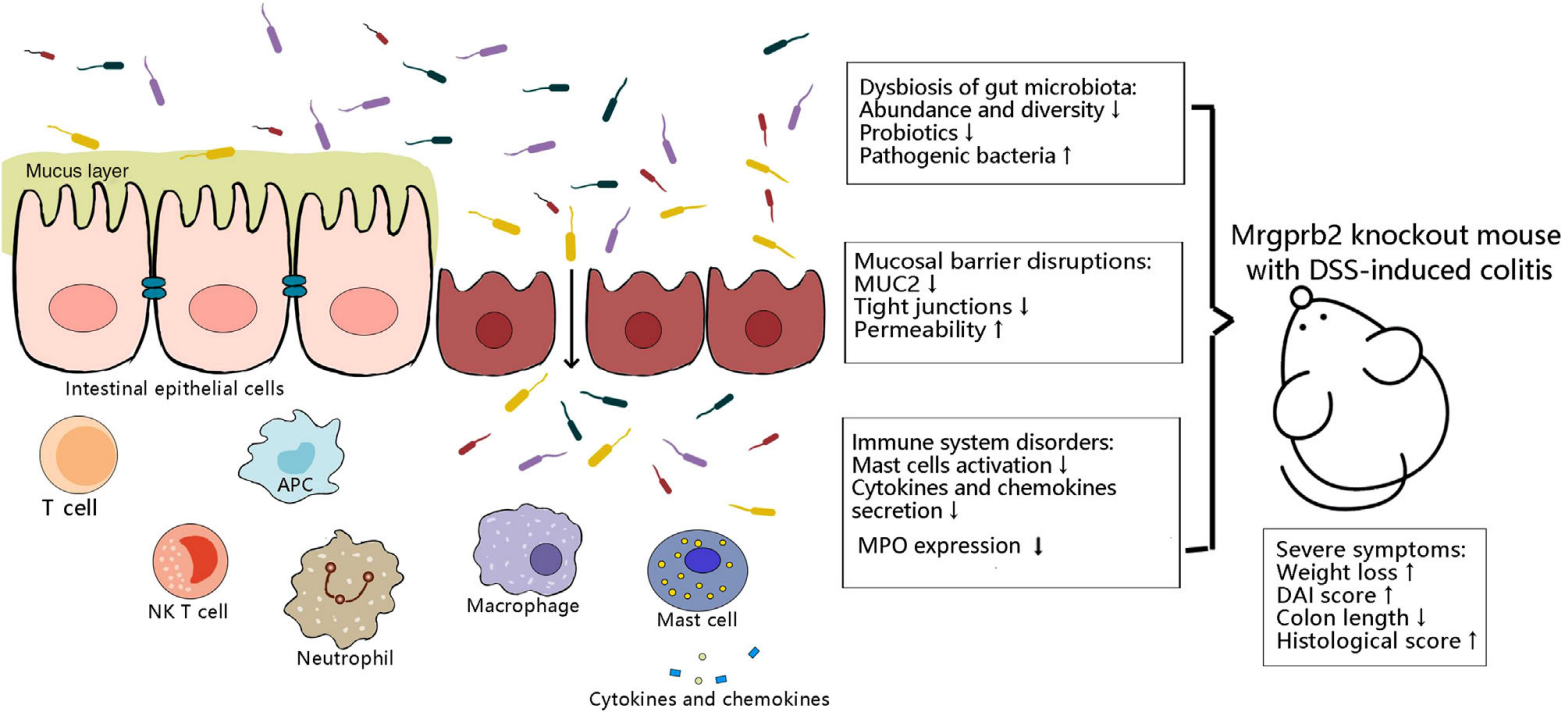
B2KO aggravated DSS‐induced colitis by exacerbating gut microbiota dysbiosis, mucosal barrier disruption and immune system disorder

MCs are constitutively found in the gastrointestinal tract and are thought to be associated with many intestinal diseases including UC, IBS and food allergies (Wouters et al., 2016). MRGPRX2/Mrgprb2 is a receptor specifically expressed on MCs (but also dorsal root ganglion cells) and mediates degranulation of MCs (Redegeld et al., 2018). We constructed a b2KO mouse model and treated these mice with DSS to induce colitis and found that the b2KO mice showed more severe symptoms. MC activation was attenuated because of the lack of Mrgprb2. Cytokines and chemokines secreted by MCs were reduced, resulting in less infiltration of phagocytic immune cells. On the other hand, the abundance and diversity of intestinal flora in b2KO mice declined, the growth of pathogenic bacteria increased while the proportion of beneficial bacteria decreased, and severe destruction of the intestinal barrier made it easier for pathogens to invade (Figure 7). These results suggested that the presence of Mrgprb2 might have a protective effect on mice with colitis.

MCs are one of the key effector cells in the inflammatory process (Forsythe, 2019), and MRGPRX2/Mrgprb2 plays an important role in MC activation. The loss of Mrgprb2 on MCs leads to a reduction in their activation, fewer cytokines secreted and inflammatory cell recruitment. In our model of colitis we found the expression of chemokines such as CCL2 and CCL3 and infiltration of neutrophils was decreased in b2KO mice. Less histamine and TNF‐α release from MCs results in weak dendritic cell activation resulting in attenuated antigen presentation to T cells (Subramanian et al., 2016). Reduced infiltration and activation of innate and adaptive immune cells attenuates host resistance to pathogens. Mrgprb2 is also involved in the interaction between MCs and nerves. NGF plays a major role in neural development and stimulates MCs to release pro‐nociceptive mediators such as histamine and NGF, which support a positive feedback loop (Gupta & Harvima, 2018). In our study, b2KO mice showed reduced NGF secretion. The neuroprotective and neurotrophic properties of NGF are impaired under pathological conditions (Xu et al., 2019).

The intestinal barrier is composed of intestinal flora, intestinal mucus, intestinal mucosal epithelium and the underlying mucosal immune system (Odenwald & Turner, 2017). Goblet cells in the intestinal epithelium synthesize and secrete gel‐forming mucins. The mucin layer creates net‐like structures that bacteria cannot penetrate and provides both lubrication and physical barrier functions (Kurashima & Kiyono, 2017). One study assessed whether impaired intestinal permeability was associated with bowel symptoms of diarrhoea or abdominal pain, and found that the Confocal Leak Score (range: 0 = no impaired permeability to 100 = complete loss of barrier function) of patients with symptomatic IBD was significantly higher than in patients with asymptomatic IBD or controls, which indicated impaired intestinal permeability correlated with ongoing bowel symptoms (Kurashima & Kiyono, 2017). The inner mucus layer of humans separates bacteria from the epithelial cells, while active UC patients have a penetrable inner mucus layer that allows bacteria to reach the epithelium (Johansson et al., 2014). The main mucus component in the gut is MUC2, which is a large and heavily *O*‐glycosylated gel‐forming mucin. In patients with UC, the thickness of these mucous layers is reduced due to the loss of goblet cells, and the goblet cell secretory response to microbial challenge is attenuated (Birchenough et al., 2015). The expression of major structural mucus components including MUC2 is reduced in active UC, and MUC2‐deficient mice do not secrete any mucus, have bacteria in direct contact with the epithelium and spontaneously develop colitis (van der Post et al., 2019). TJs composed of claudins, ZO‐1 and members of the occludin family are intercellular adhesion complexes essential for epithelial and endothelial barrier function (Zeisel et al., 2019). As an important protein component of TJs, occudin plays an important role in maintaining intestinal barrier function. Mucosal biopsy showed that patients with UC exhibited lower occludin levels than the controls and the decrease was correlated with lower levels of caspase‐3 expression (Kuo et al., 2019). In our study, the expression of MUC2 and occludin in b2KO mice was decreased, which means the gut barrier was damaged, making it easier for bacteria to invade.

The microbial patterns among UC patients exhibit reduced microbial diversity, increased instability of the gut microbiota composition over time, decreased relative abundance of Firmicutes, and an increase in Proteobacteria (Lee & Chang, 2021). Reduced gut microbial diversity may result in impaired integrity of the intestinal barrier and damaged regulation of the host immune system. Mucolytic bacteria and pathogenic bacteria are also on the rise, leading to degradation of the mucosal barrier, which allows pathogens to move further into intestinal tissues (Alipour et al., 2016; Chassaing & Darfeuille‐Michaud, 2011; Ng et al., 2013). Our results showed that b2KO mice with induced colitis exhibited reduced microbial abundance and diversity. Compared with the other three groups, the abundance of Firmicutes in the b2KODSS group was significantly lower, and the abundance of Proteobacteria was elevated. At the genus level, the dominant bacteria in each group were norank_f_Muribaculaceae, and in b2KODSS group the abundance of norank_f_Muribaculaceae was strikingly reduced. Some strains of norank_f_Muribaculaceae use intestinal mucus polysaccharides as growth nutrients and can inhibit the growth and colonization of pathogenic bacteria such as *Flavobacterium difficile* in the intestine by occupying its ecological space (Pereira et al., 2020). Another probiotic in UC treatment, *Lactobacillus*, which is a SCFA generation bacterium, was related to a lower abundance of pathogenic bacteria and decreased inflammatory markers (Nascimento et al., 2020) and was lower in both DSS treatment groups. *Escherichia*–*Shigella* is a typical genus of Proteobacteria and includes lipopolysaccharide‐producing Gram‐negative bacteria. Its relative abundance in IBD patients is higher than in healthy people (Santoru et al., 2017) and there was a dramatically increased abundance of this classic pathogen in the b2KODSS group. Increased pathogenic bacteria are more likely to invade the damaged intestinal barrier and cause disease.

## 5 CONCLUSIONS

In this study we demonstrated that b2KO mice with colitis had more severe symptoms. Mrgprb2 deletion mice had altered intestinal flora and more serious damage to the intestinal barrier. The reduced infiltration of inflammatory cells attenuated the body's ability to eliminate invading pathogens and ultimately led to more severe disease. Future studies are needed to verify the specific molecular mechanism of Mrgprb2.

## AUTHOR CONTRIBUTIONS

Conceptualization, M.S.; data curation, M.S.; formal analysis, M.S.; funding acquisition, H.L.; investigation, M.S. and J.L.; methodology, M.S.; drafting of the work and revising it critically for important intellectual content, M.S., J.L. and H.L. All authors have read and approved the final version of this manuscript and agree to be accountable for all aspects of the work in ensuring that questions related to the accuracy or integrity of any part of the work are appropriately investigated and resolved. All persons designated as authors qualify for authorship, and all those who qualify for authorship are listed.

## CONFLICT OF INTEREST

The authors declare no conflict of interest.

## FUNDING INFORMATION

None.

## Supporting information



Statistical Summary Document

## Data Availability

The 16S rRNA gene sequences were deposited in the GenBank Sequence Read Archive (SRA) database (ID PRJNA812349).
